# Swiprosin-1 stimulates cancer invasion and metastasis by increasing the Rho family of GTPase signaling

**DOI:** 10.18632/oncotarget.3637

**Published:** 2015-04-11

**Authors:** Yun Hyun Huh, Sena Oh, Yu Ra Yeo, In Hee Chae, So Hee Kim, Ji Shin Lee, Sook Jung Yun, Kyu Yeong Choi, Je-Hwang Ryu, Chang-Duk Jun, Woo Keun Song

**Affiliations:** ^1^ Bio Imaging and Cell Dynamics Research Center, Gwangju Institute of Science and Technology, Gwangju, Korea; ^2^ School of Life Sciences, Gwangju Institute of Science and Technology, Gwangju, Korea; ^3^ Department of Pathology, Chonnam National University Hwasun Hospital, Hwasun, Korea; ^4^ Department of Dermatology, Chonnam National University Hospital, Gwangju, Korea; ^5^ The Division of Natural Medical Sciences, College of Health Science, Chosun University, Gwangju, Korea; ^6^ Dental Science Research Institute and Research Center for Biomineralization Disorders, School of Dentistry, Chonnam National University, Gwangju, Korea

**Keywords:** Swiprosin-1, metastasis, invasion, migration, Rho GTPase

## Abstract

Ectopic expression of Swiprosin-1, an actin-binding protein (also known as EF hand domain containing 2; EFHD2), enhanced motile protrusions associated with actin, such as lamellipodia and membrane ruffles. Swiprosin-1 levels were increased in various human cancer tissues, particularly at highly invasive stages of malignant melanoma. Expression of Swiprosin-1 was correlated with that of epidermal growth factor receptor (EGFR) and induced by EGF. In a mouse metastasis model, Swiprosin-1 overexpression induced pulmonary metastasis whereas its knockdown led to marked inhibition of metastasis of highly invasive melanoma cells. Swiprosin-1 at the lamellipodia and membrane ruffles controlled the direction of cell protrusion and enhanced migration velocity through activating the Rho family of small GTPases, including Rac1, Cdc42 and RhoA. Our collective findings support the potential utility of Swiprosin-1 as a therapeutic target to prevent cancer invasion and metastasis.

## INTRODUCTION

Invasion and metastasis remain the leading causes of cancer-related mortality. Elucidation of the mechanisms underlying acquisition of metastatic potential by cancer cells is critical for diagnosis and development of effective treatment regimes. Metastasis is a complex process that involves proliferation within tissues, expansion to adjacent tissues, and dissemination to distant organs [1]. Increased motility plays a crucial role in the metastatic cascade. Cell migration begins with the formation of motile protrusions, such as lamellipodia, filopodia and membrane ruffles containing actin and actin-associated proteins. Various regulators of the actin cytoskeleton are involved in the acquisition of invasive and metastatic phenotypes. Expression levels of actin-binding proteins, such as components of the Arp2/3 complex, N-WASP, WAVE, and Fascin, are abnormally up- or downregulated in a variety of cancer tissues and cell lines [2]. Fascin, an actin bundling protein, is upregulated in several cancer types, including thymic [3], endometrial [4], pancreatic [5], and hepatocellular [6] carcinoma, and regulates migration, invasion and MMP expression of pancreatic ductal adenocarcinoma [7]. Knockdown of ARPC2 or the ARPC5 subunit of the Arp2/3 complex has been shown to attenuate invasion of SK-BR3 breast cancer and human head-and-neck squamous carcinoma cells [8, 9]. Notably, depletion of Fascin in melanoma CHL1 or MDA-MB-231 breast adenocarcinoma cell lines leads to significant reduction in invadopodia [10] with specialized actin-rich membrane structures [11], which initiates tumor invasion.

Members of the Rho family of guanosine triphosphatases (GTPase) (RhoA, Rac1, and Cdc42) function in multiple signaling pathways leading to cell adhesion, migration, proliferation and transformation [12]. Rho GTPases contribute to metastasis through regulation of actomyosin contraction and reorganization of the actin cytoskeleton [13]. Rac1 has been identified as the third most commonly mutated protooncogene (P29S substitution) in melanoma after BRAF and NRAS [14]. Rac-WAVE2 signaling promotes invasion and metastasis of murine melanoma [15], and overexpression of LIM kinase 1 in human breast cancer cell lines increases motility through regulation of Rock and Rho signaling pathways [16]. In addition, Rac1 has been proposed as a therapeutic target for gefitinib-resistant non-small-cell lung cancer [17]. RhoA mutations have been specifically identified in diffuse-type gastric carcinoma, supporting its utility as a potential target for gastric cancer therapy [18].

Swiprosin-1 (also known as EF hand domain containing 2; EFHD2) was initially identified in human CD8+ lymphocytes [19], and subsequently in B cells [20] and non-lymphoid tissues, such as brain, lung, and spleen [21]. A number of reports support an association between Swiprosin-1 and F-actin reorganization. Swiprosin-1 accumulation has been reported in actin cytoskeleton-rich regions in HMC-1 human mast cells [22]. Moreover, Swiprosin-1 fractionates with actin and actin-binding proteins, such as filamin, plastin, and α-actinin, in the cytoskeleton fraction in NK-like cells [23]. Recently, Swiprosin-1 was characterized as an actin-binding protein and shown to induce F-actin bundling in the presence of Ca^2+^, dependent on self-dimerization through both EF-hand motifs and the coiled-coil domain [24]. In particular, direct interactions between Swiprosin-1 and F-actin modulate membrane dynamics, such as lamellipodia formation. Overexpression of Swiprosin-1 enhances the formation of motile protrusions in B16F10 mouse melanoma [25]. In human non-small lung cancer H-460 cells, Swiprosin-1 is associated with ezrin/radixin/moesin and membrane-cytoskeleton linkers involved in cell migration and metastasis [26].

The activity of Swiprosin-1 in regulating lamellipodial membrane dynamics as an actin binding and bundling protein indicates a potential role in invasion and metastasis, although its specific function in cancer cell progression remains to be established. In the current study, we demonstrated a regulatory role of Swiprosin-1 on invasion and metastasis of melanoma, supporting its potential utility as a therapeutic target to control cancer progression.

## RESULTS

### Expression of Swiprosin-1 in cancer cell lines and human cancer tissues

**Figure 1:**
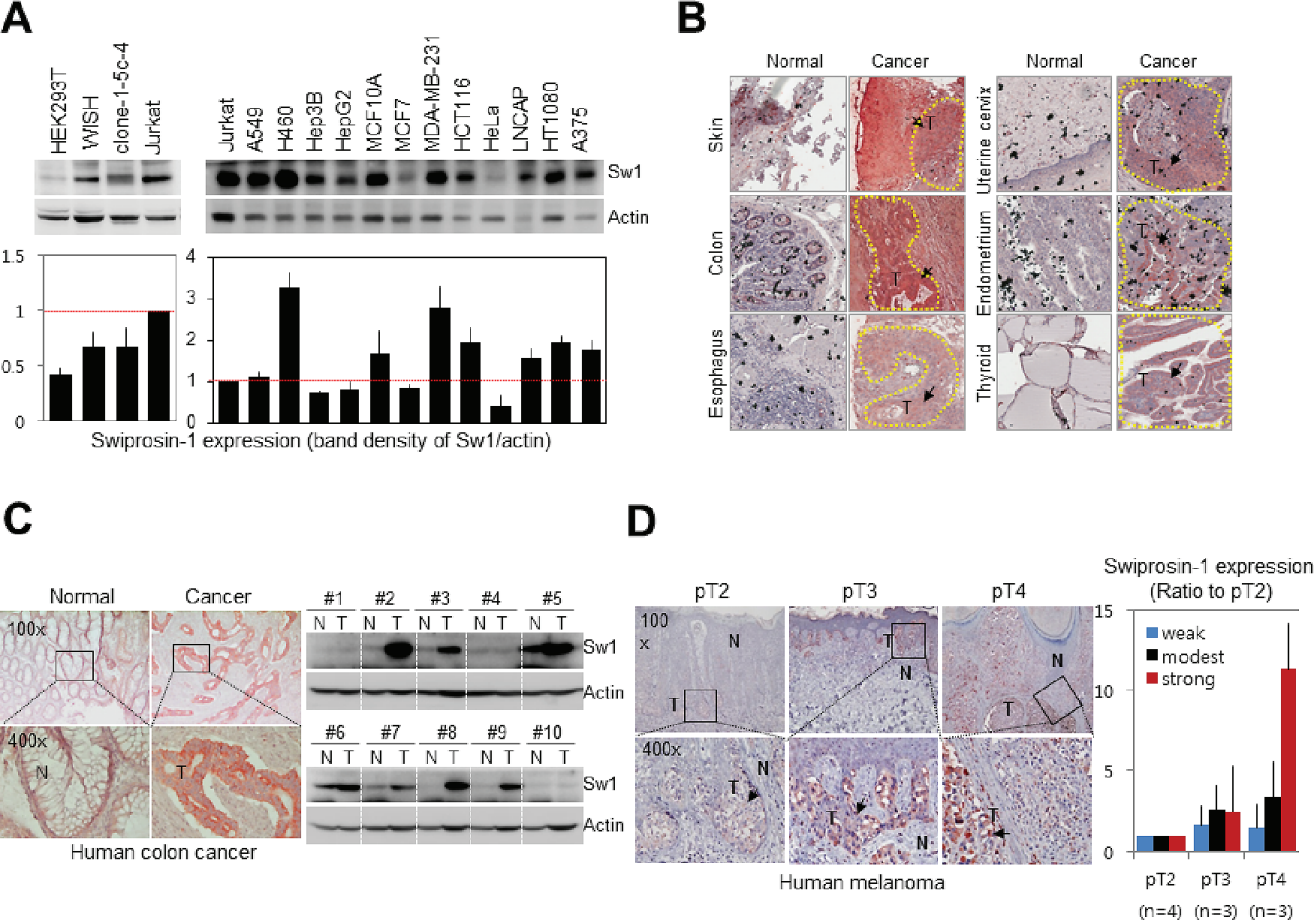
Upregulation of Swiprosin-1 in cancer cell lines and human cancer tissues **A.** Expression of Swiprosin-1 in 12 cancer and 4 normal human cell lines, including Jurkat T cells as a positive control, was determined using western blot (upper). Densitometric quantification results were obtained from three independent experiments (lower). **B.** Immunohistochemical analysis of Swiprosin-1 expression in tissue microarray containing 30 normal and 29 cancer tissue sections from human cancer patients. Representative tissues with strong Swiprosin-1 expression are shown. **C.** Human normal (N) and colorectal cancer tissues (T) were immunostained (left) and subjected to western blot (right) with anti-Swiprosin-1 antibody. Ten patients were independently assessed. A typical immunostaining result is presented. **D.** Human melanoma tissues from patients (*n* = 10) were immunostained with anti-Swiprosin-1 antibody. The intensity of positive staining was quantified using Aperio ImageScope software, and T categories classified by the American Joint Committee on Cancer Melanoma Staging.

Information on expression of Swiprosin-1 in various cancer cells was collected from various databases (http://www.genecards.org/cgi-bin/carddisp.pl?gene=EFHD2). Swiprosin-1 expression was additionally determined in a variety of human cancer cells via immunoblotting (Figure 1A) using Jurkat cells as a positive control [24]. Except MCF7 and HeLa, the majority of cancer cell lines examined displayed high Swiprosin-1 expression, while normal human cell lines, including HEK293T, WISH and clone-1-5c-4, expressed low levels of Swiprosin-1, compared with Jurkat cells (Figure 1A). In tissue microarray containing 30 normal and 29 human cancer tissue sections (supplementary Table 1), significant Swiprosin-1 expression was observed in the majority of carcinomas, including adenocarcinoma and squamous cell carcinoma (supplementary Figure S1A). Swiprosin-1 expression was markedly increased in skin, colon, esophagus, uterine cervix, endometrium and thyroid cancer tissues (Figure 1B and supplementary Figure S1B). Further analysis of colon and melanoma tissue obtained from individual patients revealed significant upregulation of Swiprosin-1 in both colon cancer (*n* = 10) (Figure 1C) and melanoma (*n* = 10) (Figure 1D). Immunohistochemical findings further disclosed specific Swiprosin-1 expression in cancer cells from tumor regions (Figure 1C and 1D, arrows). The specificity of anti-Swi-1 antibody was validated in melanoma by incubating with normal goat IgG (supplementary Figure S2). Interestingly, expression of Swiprosin-1 was dramatically increased in highly invasive cancer cells in pT4, compared to pT2 and pT3 melanoma (Figure 1D). The intensity of positive pixels (Figure 1D) was quantified using Aperio ImageScope software (Figure 1D, right panel). Our collective findings indicate that Swiprosin-1 is upregulated in a number of cancer cell lines and human cancer types (such as colon cancer and melanoma), but not all cancer tissues.

### Swiprosin-1 is upregulated through EGF signaling in melanoma

**Figure 2:**
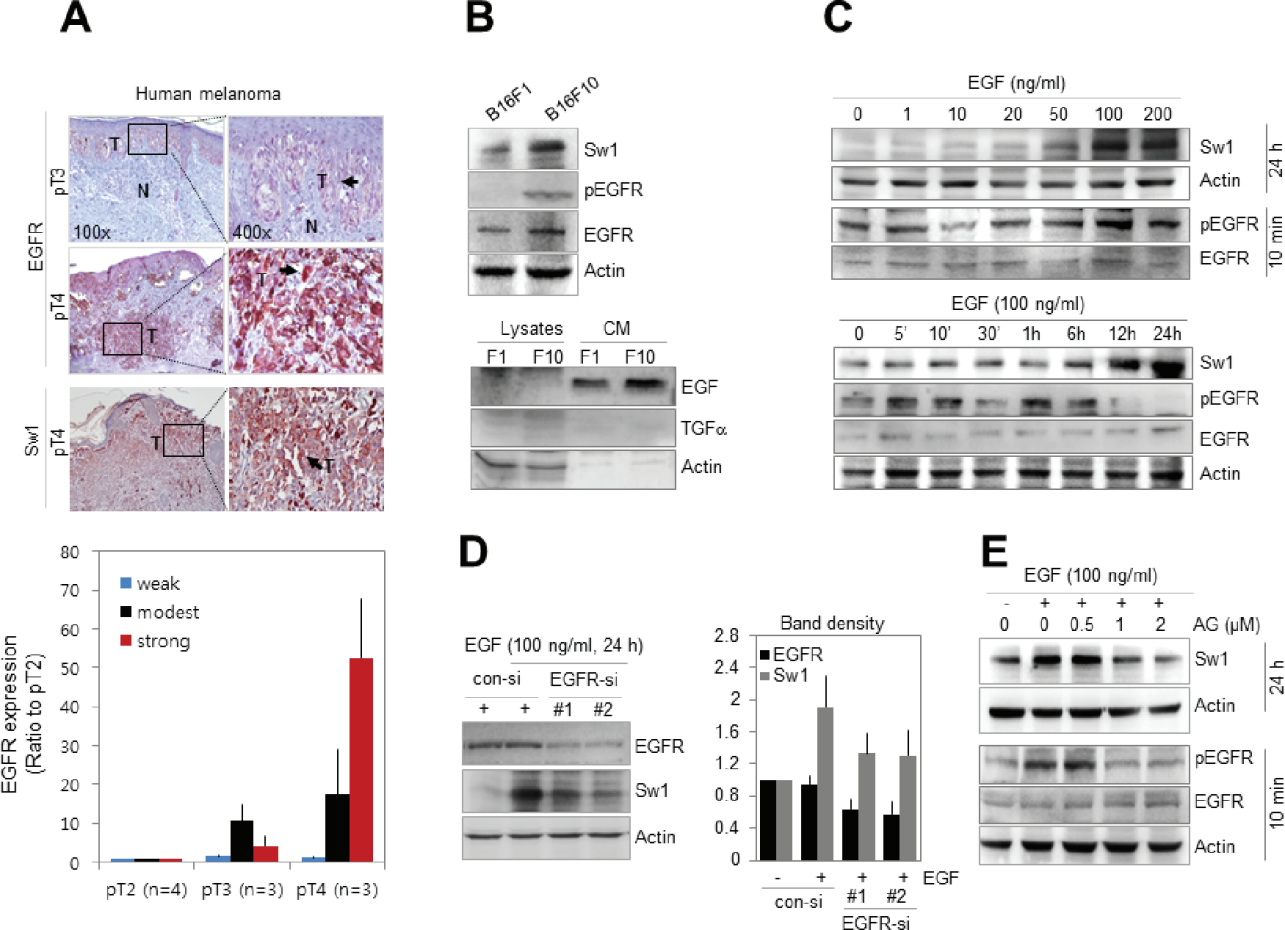
Swiprosin-1 expression is regulated by EGF signaling in melanoma **A.** EGFR and Swiprosin-1 expression patterns in human melanoma tissues (*n* = 10) were examined using immunohistochemistry and analyzed with Aperio ImageScope. **B.** Expression levels of EGFR and Swiprosin-1, and pEGFR levels in B16F1 and B16F10 cells were examined via western blot. For detection of EGF and TGFα, conditioned medium was prepared by culturing for 36 h in serum-free medium. **C.** Cells were treated with the indicated concentrations of EGF for 24 h for Swiprosin-1 expression or 10 min for EGFR phosphorylation (upper). Cells were additionally stimulated with 100 ng/ml EGF for the specified times (lower). **D.** B16F10 cells were transfected with EGFR-specific siRNA (#1 and #2) at 100 μM and treated with 100 ng/ml EGF for 24 h. EGFR and Swiprosin-1 expression levels were assessed with western blot and band densities quantitated using Multi Gauge V3.0 software. **E.** Cells were pre-treated with the indicated concentrations of AG1478, a specific antagonist of EGFR, for 1 h, and stimulated with EGF for 24 h or 10 min for detection of Swiprosin-1 expression (upper) and EGFR phosphorylation (lower), respectively.

Based on previous studies showing upregulation of EGF and EGF receptor (EGFR) in malignant melanoma [27, 28], the correlation between Swiprosin-1 expression and EGFR signaling was examined. Stronger staining for EGFR was observed at pT4 than pT3 stages of human melanoma (*n* = 10) expressing high levels of Swiprosin-1 (Figure 2A). Consistent with immunohistochemical results from human melanoma tissues, both EGFR and Swiprosin-1 were upregulated in high-metastatic mouse melanoma B16F10 cells (Figure 2B), compared to low-metastatic B16F1 cells. Notably, the phospho-EGFR (pEGFR) level was higher in B16F10 than B16F1, and EGF was detected in conditioned media of both cell lines, but not TGFα, a ligand of EGFR. Swiprosin-1 expression was increased in the presence of EGF in a dose- and time-dependent manner in B16F1 (Figure 2C) and decreased upon knockdown of EGFR using RNAi in B16F10 cells (supplementary Figure S3). EGFR knockdown additionally inhibited the increase in EGF-induced Swiprosin-1 expression in B16F1 cells (Figure 2D). Increased Swiprosin-1 expression was detected 6 h after EGF treatment and continued up to 24 h. Pre-treatment with AG1478, an antagonist of EGFR, prior to EGF stimulation, inhibited the EGF-mediated increase in Swiprosin-1 expression (Figure 2E). The antagonistic effect of AG1478 was confirmed with detection of EGFR phosphorylation (Figure 2E). Our data collectively indicate that Swiprosin-1 is upregulated via the EGFR signaling pathway in malignant melanoma.

### Swiprosin-1 expression regulates pulmonary metastasis of B16F10 melanoma

**Figure 3:**
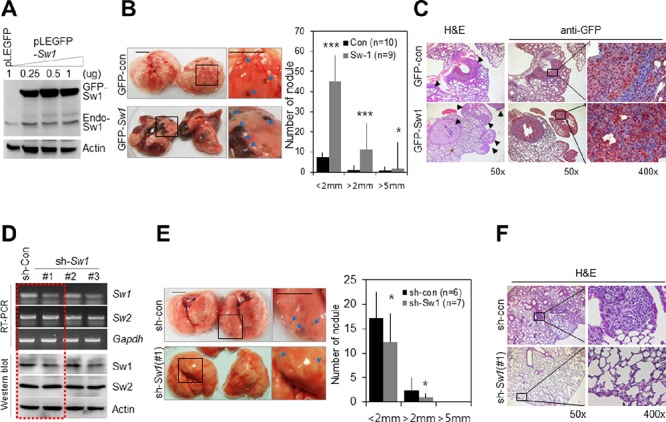
Swiprosin-1 regulates metastasis of B16F10 melanoma *in vivo* **A.** Overexpression of GFP-Swiprosin-1 in B16F10 cells was verified by western blot with anti-GFP and anti-Swiprosin-1 antibodies. **B.** and **C.** B16F10 cells (2 × 10^5^ cells in PBS) stably expressing GFP-Swiprosin-1(GFP-Sw1) or GFP-control (GFP-con) were injected into the tail veins of C57BL6 mice. After two weeks, the sizes and numbers of black nodules (blue arrows) in lung were counted, *Bar*, 5 mm (B) Paraffin sections of mouse lungs were prepared, and metastatic foci derived from GFP-control or GFP-Swiprosin-1-expressing cells stained with H&E or anti-GFP antibody. Arrowheads indicate pulmonary metastases (C) **D.** Knockdown of Swiprosin-1 with specific shRNAs (#1, #2 or #3) in B16F10 cells, determined using RT-PCR and Western blot. **E.** and **F.** Intravenous injection of B16F10 cells stably transduced with shRNA-Swiprosin-1 [sh-Sw1 (#1)] and shRNA-control (sh-con). Representative images of lungs with black nodules. The numbers of black nodules (blue arrows) were counted and sizes of nodules classified according to diameter, *Bar*, 5 mm (E). Paraffin sections of pulmonary tissue were stained with H&E (F).

Next, we examined the effects of overexpression or knockdown of Swiprosin-1 on *in vivo* metastasis. Overexpression of GFP-Swiprosin-1 in B16F10 cells was detected using immunoblotting (Figure 3A), and cells stably expressing GFP-Swiprosin-1 established with neomycin. Injection of mice with B16F10 cells stably expressing GFP-Swiprosin-1 resulted in the appearance of black nodules in lung, indicative of pulmonary metastases. The number (~4 times) and size of black nodules were significantly increased in mice injected with B16F10 cells stably expressing GFP-Swiprosin-1, compared to those injected with GFP-control (Figure 3B). H&E staining and immunohistochemical analysis with anti-GFP antibody (Figure 3C) verified that the observed pulmonary nodules (arrows in Figure 3C) were derived from injected B16F10 cells stably expressing GFP-Swiprosin-1. To examine the knockdown effect of Swiprosin-1 on metastasis of melanoma, shRNA targeting Swiprosin-1 regions conserved in both human and mouse (sh#1) or specific for either human (sh#2) or mouse (sh#3) sequences were designed, and their silencing efficiencies verified using RT-PCR and immunoblotting. Swiprosin-1 shRNA had no effect on expression of Swiprosin-2, an isoform of Swiprosin-1 with over 70% amino acid sequence homology (Figure 3D). When B16F10 cells stably transduced with the most efficient shRNA-Swiprosin-1 (#1) were injected into mice, pulmonary metastasis was dramatically inhibited (Figure 3E), showing reduced size and number of pulmonary nodules (Figure 3F). Our results indicate that Swiprosin-1 levels regulate pulmonary metastasis of melanoma.

### Swiprosin-1 promotes invasion of B16F10 cells by enhancing cell motility

**Figure 4:**
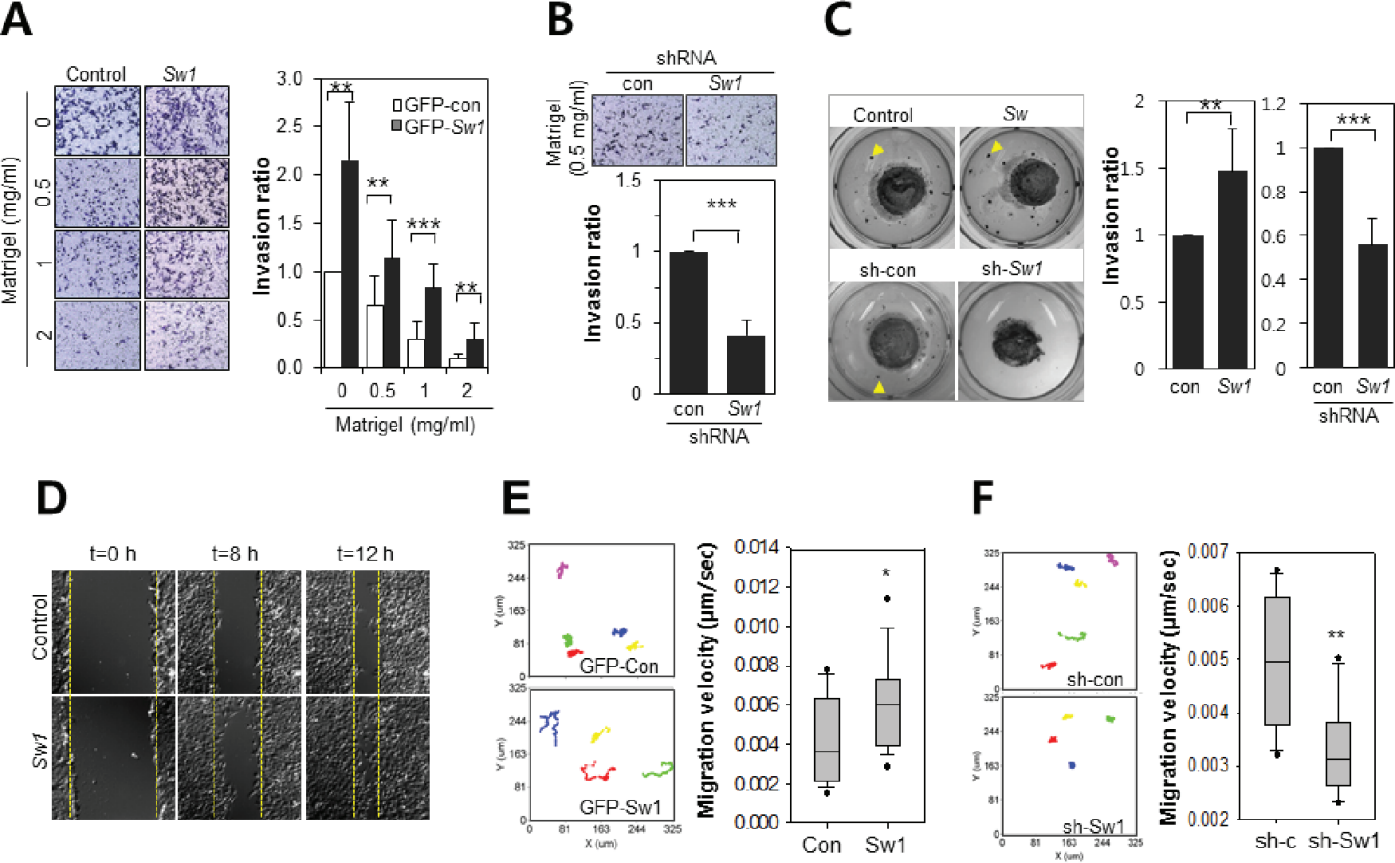
Swiprosin-1 enhances the invasion of B16F10 melanoma cells **A.** and **B.** The matrigel invasion assay was performed with B16F10 cells stably expressing GFP-control or GFP-Swiprosin-1(Sw1) (A), and cells stably transduced with shRNA-control (sh-con) or shRNA-Swiprosin-1 (sh-Sw1, #1) (B) The transwell membrane was coated with matrigel (0.5 mg/ml). Cells invading the matrigel-coated membrane were stained with crystal violet (up), and counted on nine randomly selected membrane areas (*** < 0.001, lower panel). **C.** 3D cell culture assay using collagen gel was performed. B16F10 cells stably expressing GFP-control or GFP-Swiprosin-1(Sw1) and those stably transduced with shRNA-control (sh-con) or shRNA-Swiprosin-1 (#1, sh-Sw1) were embedded in collagen gel and soaked in matrigel. Gels were subsequently covered with fibrin gels. After 14 days, migrating satellite colonies in the fibrin matrix were stained with methylene blue and quantified (** < 0.05, *** < 0.001). **D.** Relative migration of cells stably expressing GFP-control or GFP-Swiprosin-1 with the wound healing assay was assessed by scraping the monolayer and observing wound closure. Time-lapse images were obtained every 20 min for 12 h. **E.** and **F.** B16F10 cells stably expressing GFP or GFP-Swiprosin-1 (E) and stably transduced with shRNA-Swiprosin-1 (#1) and shRNA-control were cultured (F) Time-lapse images were captured every 10 min for 9 h (*n* > 20 cells, *P < 0.05, **P < 0.005) and analyzed by tracking nuclear positions and velocities of cell migration using Metamorph software. See supplementary information, Movie SM1.

To confirm the regulatory role of Swiprosin-1 in the metastasis of B16F10 melanoma, an *in vitro* modified Boyden chamber assay and *in vivo*-like 3-D collagen gel assay were performed. As shown in Figure 4A, the invasive activity of Swiprosin-1-overexpressing cells was maintained at a high matrigel concentration (2 mg/ml), but almost abolished in GFP-control B16F10 cells (*p* < 0.05). Upon transduction of B16F10 cells with shRNA-Swiprosin-1(#1), invasive activity was significantly inhibited, even at low concentrations of matrigel (0.5 mg/ml) (Figure 4B). In the *in vivo*-like 3-D collagen culture assay, colony size and distance from collagen gel were remarkably increased in Swiprosin-1-overexpressing B16F10 cells (Figure 4C, upper). In contrast, few satellite colonies were generated upon knockdown of Swiprosin-1 (Figure 4C, lower). Cells overexpressing Swiprosin-1 showed rapid wound closure in wound-healing assays (Figure 4D) and live time-lapse imaging analysis for 9 h, supporting a regulatory role on cell motility. Moreover, Swiprosin-1 overexpressing cells moved more rapidly (0.00656 ± 0.00188 μm/s) than control cells (0.00409 ± 0.00212 μm/s), and tended to travel in a straight line, while control cells generally congregated around the original position (Figure 4E and supplementary movie SM1). Knockdown of Swiprosin-1 led to dramatic reduction of cell motility (Figure 4F). Accordingly, we propose that Swiprosin-1 promotes invasion of cancer cells by enhancing cell motility.

### Swiprosin-1 induces motile protrusions associated with actin

**Figure 5:**
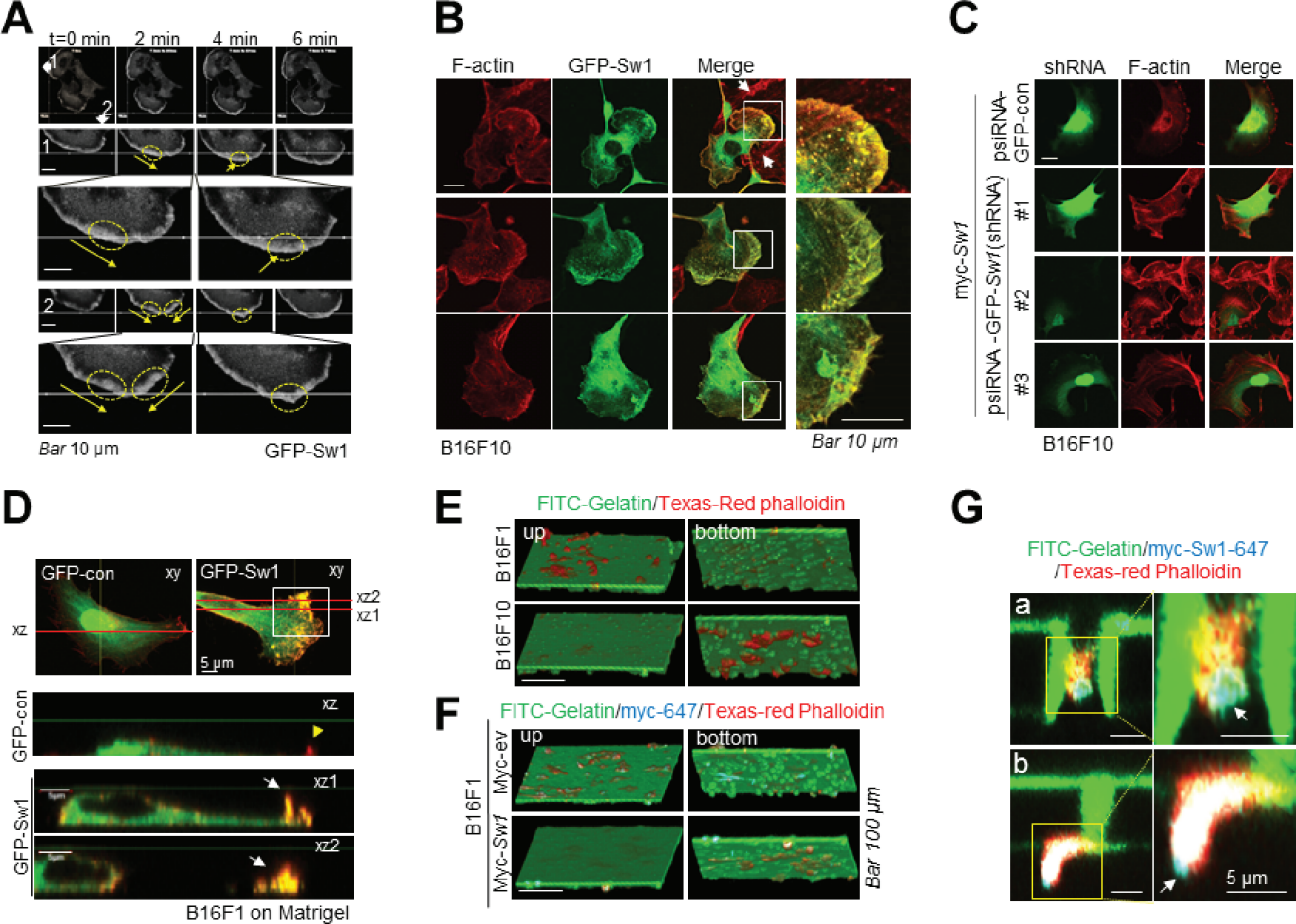
Swiprosin-1 induces the formation of motile protrusions associated with actin **A.** Time-lapse fluorescence images were captured at 5 sec intervals for 10 min under a confocal microscope. White arrowheads indicate the direction of movement, yellow arrows represent direction of GFP-Swiprosin-1 movement and yellow circles signify accumulated Swiprosin-1 and protrusion. *Bar*, 10 μm. See supplementary information, Movie SM2. **B.** B16F10 cells stably expressing GFP-control or GFP-Swiprosin-1 (green) were stained with Alexa594-phalloidin (red) to visualize F-actin. Merged images are shown in yellow. Scale bar, 10 μm. Lamellipodia, microspikes and membrane ruffles were observed in B16F10 cells stably expressing GFP-Swiprosin-1 (*n* > 30 cells). **C.** B16F10 cells stably transduced with shRNA-control or shRNA Swiprosin-1 (sh-Sw1) were transfected with myc-Swiprosin-1. After incubation for 24 h, cells were stained with Alexa594-phalloidin. Significant formation of membrane protrusions was observed in cells transduced with shRNA-control, but not sh-Sw-1. **D.** B16F1 cells on matrigel were transfected with GFP-control or GFP-Swiprosin-1. Vertical section images of the cell (along the lines in merged images) are shown below horizontal confocal images. White arrows indicate aberrant ruffles on the dorsal side of cells and the yellow arrowhead signifies peripheral ruffles at the cell edge. **E.** Cells were cultured for 3 h on the upper layer of the transwell membrane coated with FITC-conjugated gelatin, and the transwell membrane cut and stained with Texas red-conjugated phalloidin, followed by confocal microscopy. Serial vertical section images of cells on the transwell membrane are shown in 3D. **F.** and **G.** B16F1 cells were transfected with Myc-tagged Swiprosin-1 (Myc-Sw1) or empty vector (ev) and seeded on FITC-conjugated gelatin-coated Transwell. After 3 h, cells were stained with anti-myc antibody and Alexa 647-conjugated anti-mouse secondary antibody, followed by Texas Red-conjugated phalloidin. Vertical section images were obtained by sequential scanning under a confocal microscope to eliminate cross-talk from triple-stained samples. *Bar*, 100 μm. Higher magnification images showing the tips of moving cells through pores of transwell (G) *Bar*, 5 μm. See supplementary information, Movies SM3 and SM4.

Live imaging analysis disclosed rapid translocation of Swiprosin-1 to the leading edges of motile cells (arrow in Figure 5A and Supplementary movie SM2). Membrane protrusions were evident at the sites of Swiprosin-1 accumulation (yellow circle in Figure 5A) and cells moved rapidly towards the direction of the protrusion. Consistent with previous findings [24, 25] Swiprosin-1 induced membrane ruffles, microspikes, as well as lamellipodia, and co-localized with F-actin (Figure 5B). Knockdown of Swiprosin-1 with shRNA (#1 and #3) abrogated Swiprosin-1-induced lamellipodia formation (Figure 5C). B16F10 cells display numerous ruffle structures towards the dorsal side, including aberrant and peripheral ruffles that induce high motility [15]. Overexpression of swiprosin-1 significantly enhanced the formation of aberrant ruffles (white arrows) on the dorsal surface of B16F1 cells (Figure 5D). Invasive morphology of motile protrusion cells cultured on a transwell coated with FITC-conjugated gelatin was visualized. In vertical scanned 3D images obtained using confocal microscopy, the majority of B16F10 cells passed through pores of transwell filters and adhered to the bottom layer of the insert, while most B16F1 cells remained on the upper layer of gelatin (Figure 5E and Supplementary movie SM3). Invasion of B16F1 cells ectopically expressing myc-tagged Swiprosin-1 was enhanced to a similar extent as that of B16F10 cells (Figure 5F and Supplementary movie SM4). Enlarged images revealed that Swiprosin-1 co-localizes with actin in the tips of the leading edges of motile cells passing through (Figure 5G-a) and out of (Figure 5G-b) pores of the transwell. Swiprosin-1 (cyan blue) was detected in the tips of the leading edges of motile cells, supporting a role in determining the direction of movement. Our data suggest that swiprosin-1 modulates the invasiveness of melanoma by mediating the formation of motile protrusions in association with actin.

### Swiprosin-1-induced motile protrusions are associated with the Rho family of GTPases

**Figure 6:**
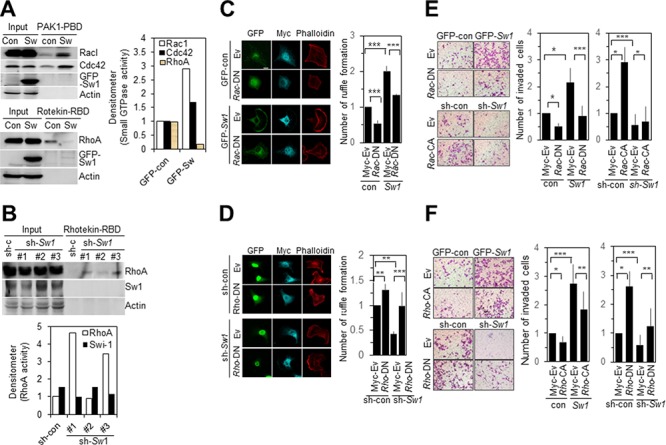
Swiprosin-1 regulates the invasion of B16F10 cells via modulating Rac1 and RhoA activities **A.** and **B.** Activities of the Rho family of GTPases were analyzed with the pulldown assay in B16F10 cells stably expressing GFP-control or GFP-Swiprosin-1 (A), and cells transduced with shRNA-control or shRNA-Swiprosin-1 (B) Rac1-GTP and Cdc42-GTP were pulled down with GST-fused Pak1-PBD, and RhoA-GTP with GST-fused Rhotekin-RBD. **C.** and **D.** B16F10 cells stably expressing GFP-control or GFP-Swiprosin-1 (C) and cells stably transduced with shRNA-control or shRNA-Swiprosin-1 (D) were transfected with myc-RacT17N (DN) or myc-RhoT19N(DN), respectively. Cells were immunostained with anti-myc antibody (Cyan blue) and Alexa 594-Phalloidin (Red). *Bar*, 10 μm. Ruffle formation was determined with actin staining (*n* > 20, * < 0.05, ** < 0.005). **E.** and **F.** B16F10 cells stably expressing Swiprosin-1 cells were transfected with myc-tagged Rac-DN or Rac-CA (E) and Rho-CA or Rho-DN (F), and invasive activity measured by counting cells on the bottom layer of the transwell membrane. (*P < 0.05, **P < 0.005, ***P < 0.001).

Activities of members of the Rho family of GTPases were examined in B16F10 cells with overexpression or knockdown of Swiprosin-1. Overexpression of Swiprosin-1 led to enhanced activities of Rac1 and Cdc42, but reduced RhoA activity, as determined with the GST-fused PAK1-PBD or Rhotekin-RBD pulldown assay (Figure 6A). Upon suppression of Swiprosin-1 expression with specific shRNA (#1 and #3), RhoA activity was increased (Figure 6B), clearly indicating that swiprosin-1 modulates activities of the Rho family of GTPases. In parallel experiments, ectopic expression of a dominant-negative mutant of Rac1 (Rac1-DN, T17NRac1) in Swiprosin-1-overexpressing B16F10 cells significantly inhibited both lamellipodia and ruffle formation (Figure 6C, left). Ruffle formation was evaluated via phalloidin staining in cells expressing both GFP and myc-tagged Rac1 mutants (Figure 6C, right). Ectopic expression of RhoA-DN (T19NRhoA) in Swiprosin-1 knockdown cells was sufficient to rescue ruffle formation (Figure 6D). Consistent with immunofluorescence staining data, ectopic expression of Rac1-DN in Swiprosin-1-overexpressing B16F10 cells resulted in decreased invasiveness (Figure 6E), and constitutively active mutants of RhoA (RhoA-CA, G14VRhoA) inhibited Swiprosin-1-induced invasion of B16F10 cells (Figure 6F). Conversely, expression of Rac1-CA (G12VRac1) or RhoA-DN in Swiprosin-1 knockdown cells rescued invasiveness of B16F10 cells up to the control level (Figure 6E and 6F). Our data suggest that swiprosin-1 modulates invasion and metastasis of melanoma by mediating the formation of motile protrusions in association with actin and the Rho family of GTPase.

## DISCUSSION

To our knowledge, this is the first report to show upregulation of Swiprosin-1 in cancer tissues and its promotion of invasion and metastasis. Cancer metastasis is a complex process that involves detachment of cancer cells from primary tumors and entry into the circulatory system, followed by migration and invasion. These processes are initiated with the formation of motile protrusions containing actin, and depend on regulation of dynamic actin reorganization. Previous experiments by our group have shown that Swiprosin-1 is an actin binding and bundling protein, and a novel regulator of membrane dynamics, cell spreading and migration [24, 25]. In this study, we observed upregulation of Swiprosin-1 in a variety of cancer cell lines and human cancer tissues, in particular, colorectal carcinoma and malignant melanoma. Overexpression or knockdown of Swiprosin-1 modulated migration and invasion of B16F10 melanoma cells through Rac1 and RhoA signaling pathways.

Data mining performed using bioinformatics databases revealed high expression of Swiprosin-1 in a variety of cancer tissues, including skin, colon, esophagus, uterine cervix, endometrium, and thyroid cancers, but not all the cancer tissues examined. Interestingly, modest or intense expression of Swiprosin-1 was detected in most carcinomas, including adenocarcinoma and squamous cell carcinoma (supplementary Figure S1). The involvement of Swiprosin-1 in cancer invasion and metastasis was evident from data showing higher expression in pT4 melanoma than local invasive pT2 (Figure 1D). The association of Swiprosin-1 upregulation with melanoma progression suggests that expression of this protein may be related to progression of other epithelial origin cancers.

Metastasis and invasion of melanoma were modulated in a Swiprosin-1 expression-dependent manner, as confirmed with the *in vitro* matrigel-coated transwell invasion assay and *in vivo*-like 3D-collagen culture system. An *in vivo* experimental mouse model mimicking metastatic events further confirmed the functional role of Swiprosin-1 in cancer metastasis. Live imaging data (supplementary movie SM2 and Figure 5A) provided direct evidence of regulation of motile protrusion formation by Swiprosin-1. Translocation and accumulation of Swiprosin-1 to the leading edges of motile cells determines the direction of migration by facilitating the formation of motile protrusions, such as lamellipodia. In addition, Swiprosin-1 detected in the tips of leading edges of cells passing through pores of the transwell in 3D vertical-sectioned images (Figure 5G) suggest a function in controlling the direction of cell movement. Interestingly, we observed aberrant ruffling formation in B16F1 cells transfected with Swiprosin-1, which was not evident in control cells (Figure 5D). Migrating cells have polarized morphology in which a ruffle structure develops toward the direction of movement, and polarized ruffles are subdivided into peripheral and aberrant ruffles [15]. Highly malignant B16F10 cells display more aberrant ruffles towards the dorsal side of cells than B16F1 cells with low malignancy, and enhanced motility may be attributed to increased ruffling [15]. Swiprosin-1 colocalized with F-actin in both peripheral and aberrant ruffles, and overexpression of Swiprosin-1 induced aberrant ruffle formation, even in B16F1 cells cultured on matrigel. Membrane protrusions including membrane ruffles and lamellipodia are accompanied by remodeling of F-actin cytoskeleton. In membrane protrusions, cytoskeleton remodeling is under control of various actin binding proteins including cofilin, a well-known regulator of actin dynamics. The severing activity of cofilin increases the availability of free barbed F-actin ends followed by G-actin monomers, consequently leading actin polymerization. In previous our study [25], EGF-induced Swiprosin-1 phosphorylation on Ser183 residue promoted translocation of cofilin to membrane protrusion, thereby caused increase of actin-free barbed ends at the leading edges of motile cells. In accord with this observation, recent report demonstrated that the phosphoryled cortactin by EGF or IGF-1 signals releases cofilin and generates actin-free barbed ends, which promotes actin polymerization and invadopodia assembly and maturation [29–31]. Taking together, these findings support that Swiprosin-1 as a modulator of F-actin cytoskeleton may be associated with malignancy of melanoma. Notably, Swiprosin-1 did not modulate cell proliferation and MMP expression, which are key enzymes required for invading adjacent tissues (supplementary Figure S4). Fascin, an actin bundling protein, is a well defined modulator of cancer invasion and metastasis [7, 10]. Overexpression of Fascin in cancer tissues, such as breast, pancreatic and endometrial carcinoma, has been reported [3–6], that regulates MMP expression as well as cell motility [7]. Specific Swiprosin-1-mediated regulation of cell motility but not MMP expression and proliferation is a significant finding of this study. Regulation of Swiprosin-1 expression prevented cancer invasion and metastasis (Figures 3 and 4) without affecting MMP expression. Our data provide new insights into the underlying mechanisms of Swiprosin-1 action and support its utility as a potent therapeutic target for controlling the metastatic process.

The involvement of the Rho family of GTPases in motility and invasive phenotypes of cancer cells is well documented [32]. During cell migration, Rac1 is required for formation of lamellipodia and membrane ruffling at the leading edges of migrating cells [32] and CDC42 is suggested to be involved in the regulation of cell polarity, controlling the direction but not directly required for cell movement [33]. Overexpression of Swiprosin-1 enhanced membrane dynamics, including formation of microspikes at the leading edges as well as lamellipodia and membrane ruffles in which swiprosin-1 colocalized with F-actin. As shown in Figure 5A and Supplemental movie SM2, translocation of GFP-Swiprosin-1 to microspikes occurred prior to lamellipodial movement. One possibility to explain this finding is that Swiprosin-1-induced CDC42 activation modulates microspike formation at the leading edges of migrating cells and controls the direction of cell movement. Although RhoA is known to regulate the assembly of actin stress fibers and associated focal adhesion [34], the role of Rho in cell motility is a controversial issue. An earlier study showed that Rho drives cell body translocation and rear retraction [35]. In contrast, another report demonstrated that RhoA negatively influences cell migration by increasing stress fiber formation and adhesion to the substrate [36]. Our finding that reduced migration velocity and invasive activity upon knockdown of Swiprosin-1 is rescued by RhoA-DN (G14VRhoA) supports a negative function of RhoA in cancer cell movement and invasion.

Results from the current study indicate that Swiprosin-1 expression is regulated by EGFR signaling (Figure 2). EGFR (ErbB1), a member of the ErbB family, is a potentially important factor in the pathogenesis of malignant melanoma [37, 38]. EGFR expression has been reported in up to 96% of primary melanomas and 90% of metastatic lesions [39]. The EGFR signaling pathway is an important target in anticancer drug development, in view of its role in regulation of tumor cell proliferation and apoptosis [38]. Gefitinib (Iressa, AstraZeneca), a small reversible EGFR inhibitor, and IMC-C225 (Erbitux, ImClone Systems), an inhibitory anti-EGFR monoclonal antibody, have been approved for non small-cell lung and metastatic colorectal cancer treatment, respectively. In melanoma, the autocrine and paracrine actions of EGF and EGFR contribute to tumor cell proliferation and migration [27]. In an earlier study, knockdown of EGF led to markedly reduced migration and *in vivo* lymph node metastasis in melanoma cells expressing high levels of EGF [28]. Here, we demonstrated that expression of Swiprosin-1 is regulated by EGFR signaling (Figure 2) and closely correlated with that of EGFR in human melanoma tissues. The invasiveness of B16F1 and B16F10 mouse melanoma cells appeared highly dependent on Swiprosin-1 expression, and its knockdown completely inhibited invasion and metastasis, both *in vitro* and *in vivo* (Figures 3 and 4). EGFR operates through major signal transduction pathways, such as PI3K/Akt, Jak/Stat and MAPK. Swiprosin-1 expression has been shown to be upregulated in T cells and mast cells through the PKCθ and PKCβ1/ε pathways [40, 41]. However, the molecular mechanism underlying upregulation of EGF-mediated Swiprosin-1 expression in cancer cells requires further study.

In conclusion, Swiprosin-1 is highly expressed in carcinoma, particularly melanoma, and increases the motility and invasiveness of cancer cells through stimulating the activities of the Rho family of GTPases. In view of the collective findings, further research on therapeutic targeting of Swiprosin-1 with the aim of preventing cancer progression is warranted.

## MATERIALS AND METHODS

### Human cancer tissues and experimental mouse model

Human colorectal cancer and melanoma tissues were kindly provided by the Korea Biobank Network (07SA2012013-001). During colon cancer operations, tumors were surgically removed along with adjacent normal tissues around 5 cm apart. Tissue microarray slides of human cancer patients were purchased from Superbiochips Laboratory (Seoul, Korea). The Review Board of the Gwangju Institute of Science and Technology approved the use of these materials (GIST-2011-1). For the *in vivo* metastasis model, 57BL6 mice at 8–12 weeks of age were used. All animal procedures were performed with the approval of Animal Care and Ethics Committees of Gwangju Institute of Science and Technology.

### Cell culture and stable cell lines

A549, H460, Hep3B, HepG2, MCF10A, MCF7, HCT116, HeLa, HT1080, A375, B16F1, B16F10, HEK293T and WISH (human amnion epithelial) cells were obtained from the Korean cell line bank (Seoul, Korea) and grown in Dulbecco's modified Eagle's medium (DMEM; Gibco-BRL, Grand Island, NY). Jurkat (kindly supplied by Dr. Chang-Duk Jun), MDA-MB-231, LNCAP and Clone 1-5c-4 (normal human conjunctival epithelial) cells (from the Korean cell line bank) were grown in RPMI1640 (Gibco-BRL) supplemented with 10% (v/v) fetal bovine serum, 50 μg/ml streptomycin and 50 units/ml penicillin. GFP-Swiprosin-1-expressing cells were stabilized by selection with neomycin after transfection with *pLegfp-Swiprosin-1*, and Swiprosin-1-depleted cell lines generated by selection with zeocin after transfection with psiRNA-hH1GFPzeo-*Swiprosin-1*.

### Reverse transcription-polymerase chain reaction (RT-PCR)

Total RNA was isolated with TRI reagent (Molecular Research Center, Cincinnati, OH) and reverse-transcribed using TOPscript RT DryMIX (Enzynomics, Daejeon, Korea). The resulting cDNA was subjected to PCR using *Taq* polymerase (iNtRON BioTechnology, Seongnam, Korea) with the following primers: 5′-cggcagggatggcttcat-3′ (sense) and 5′-ttggcacccttaacgccc-3′ (antisense) for *Swiprosin-1*, 5′-tcttcaatccctacaccg-3′ (sense) and 5′-tggaaaatgagcaggaac-3′ (antisense) for *Swiprosin-2*, and 5′-tcaccatcttccaggagcga-3′ (sense) and 5′-cacaatgccgaagtggtcgt-3′ (antisense) for *glyceraldehyde-3-phosphate dehydrogenase (Gapdh)*.

### Immunoblotting

Cells were lysed in modified radioimmunoprecipitation assay buffer, and lysates resolved using SDS-PAGE. Gels were transferred to polyvinylidene fluoride membrane, followed by blotting with primary antibodies, including anti-Swiprosin-1 (Imgenex, San Diego, CA), anti-Rac1 (BD Biosciences, San Jose, CA), anti-RhoA (Cytoskeleton, Denver, CO), anti-Myc (Cell Signaling Technology, Boston, MA), anti-Cdc42, anti-Swiprosin-2, anti-pEGFR, anti-EGFR and anti-Actin (Santa Cruz Biotechnology, Dallas, TX) antibodies.

### RNAi transfection

Mouse EGFR-specific siRNAs designed by Ambion (Life Technologies Ltd) (#1, 5′GGAAAUAAC AGGCUUUUUGtt; #2, 5′GGAAAAGAAAGUCUGCC AAtt 3′) target a sequence starting at nucleotide 1485 lying at the junction of exons 10 and 11 (#1) and nucleotide 366 at the junction of exons 1 and 2 (#2) (ref. NM_007912.4), respectively. A proprietary siRNA sequence that does not correspond to any eukaryotic gene was used as negative control siRNA. Transfection was performed by mixing siRNA with Lipofectamine™ 2000 (Invitrogen) at a final volume of 100 μl OPTI-MEM, including 10% serum without antibiotics. The procedure was performed according to the manufacturer's instructions.

### Immunofluorescence staining and confocal imaging analysis

Cells cultured on FN-coated glass covers were fixed with 4% paraformaldehyde, permeabilized with 0.1% TritonX-100 and incubated with primary antibodies, followed by Alexa 488-conjugated secondary antibody (Molecular Probes, Eugene, OR). Actin cytoskeleton was visualized using either Alexa 555- (Molecular Probes) or Texas red (Sigma)-conjugated phalloidin. Fluorescence images were obtained using an Olympus confocal microscope (FV1000) with FV10-MSASW software. To assess migration of individual cells, 1×10^4^ cells adapted to microscopic recording media (phenol red-free DMEM containing 5 μM mitomycin C and 15 mM HEPES) were plated on a cover glass in an incubation chamber. Time-lapse movies were recorded over a period of 9 h (Figure 4E and 4F, and supplementary movie SM1) and each frame acquired every 10 min using a microMax cooled EMCCD camera mounted on a Olympus IX81 microscope (Olympus, Tokyo, Japan). Migration paths were traced from nuclear positions, and velocity calculated using Metamorph software (Ver. 6.3r6, Molecular Devices LLC, Sunnyvale, CA). Cellular translocation of GFP-Swiprosin-1 to the lamellipodial leading edge was observed by recording for 10 min every 5 sec (Figure 5A and supplementary movie SM2) using confocal microscopy.

### Cell migration and invasion assay

For the transwell migration and invasion assay, 2.5×10^4^ cells were seeded on the membrane of inserts with 8.0 μm pores (Corning Costar, Tewksbury, MA) and the lower chamber filled with NIH/3T3-conditioned medium, which served as the chemoattractant. NIH/3T3-conditioned medium was prepared by culturing confluent NIH/3T3 fibroblasts for 24 h in DMEM. For the invasion assay, inserts of the transwell were pre-coated with matrigel (BD Bioscience) or FITC-conjugated gelatin (Molecular Probes). For the wound healing assay, cells were plated on glass bottom dishes and grown to confluence. Wounds were created by scraping across the cell monolayer. The cell culture medium was substituted with fresh medium containing mitomycin C, and confocal images captured every 20 min for 12 h.

### *In vivo*-like collagen-gel matrix 3-D culture

Cells (2.5×10^4^) were mixed with collagen matrix according to the manufacturer's protocol (Millipore, Billerica, MA). Matrigel-coated collagen matrix and solutions of fibrinogen and thrombin were added to the matrix. Cells were grown for 10 to 14 days in DMEM-containing aprotinin with renewal of medium every two days. Cells in the collagen matrix were fixed in 10% formalin and stained with 0.1% methylene blue in 50% ethanol. Satellite colonies were quantified.

### RacI and RhoA activity assay

Cells were incubated with magnesium-containing lysis buffer (25 mM HEPES, 150 mM NaCl, 1% Igepal CA-630, 10% glycerol, 10 mM MgCl_2_, 1 mM EDTA). Agarose-conjugated PAK-1 PBD or Rhotekin RBD (10 μg) (Millipore, Temecula, CA) was added to cell lysates for the Rac/cdc42 and Rho assay, respectively, incubated for 1 h at 4°C, and immunoblotted with anti-Rac1, -Cdc42, -RhoA and -actin antibodies.

### Immunohistochemistry

Mouse lungs were fixed in 4% paraformaldehyde, embedded in paraffin, and sectioned at a thickness of 6 μm. Metastasis of B16F10 cells was evaluated via hematoxylin and eosin (H&E) staining. Metastasis of GFP-Swiprosin-1-overexpressing or depleted B16F10 stable cells was verified by staining with anti-GFP antibody (Santa Cruz). For immunohistochemical staining of Swiprosin-1 and EGFR, paraffin sections of human cancer tissues and TMA were deparaffinized in xylene, hydrated with ethanol, and stained using the LSAB2 horseradish peroxidase kit (Dako Co., Carpinteria, CA), according to the manufacturer's instructions. Sections on slides were incubated overnight at 4°C with antibodies against Swiprosin-1 (Imgenex: IMG-3387) or EGFR (Santa Cruz: SC-03). Next, samples were incubated with biotinylated linking and streptavidin-horseradish peroxidase reagents for 10 min. Immunoreactive proteins were visualized using a 3-amino-9-ethylcarbazole (AEC) substrate chromogen solution and counterstained with hematoxylin. Stained samples were scanned using Aperio ImageScope and analyzed with the Positive Pixel Count v.9.1 algorithm (Leica Biosystems) that computed the sum of intensity values for all weak positive pixels (IWP): weak, modest positive pixels (IP), modest and strong positive pixels (ISP), and strong, within the same area (0.44 mm^2^). Hue value was 0.05 for AEC chromogen substrate-positive color. Total intensity was calculated as the sum of IWP, IP and ISP.

### Statistical analysis

All quantified data in bar charts represent an average of results from 3–4 independent experiments as means ± standard deviation. Data in box and whisker plots represent differences between populations. The two-tailed Student *t* test with unequal variance was used for comparison of parameters between two groups. All *P* values are two-sided. Data were considered statistically significant at *P* values less than 0.05.

## SUPPLEMENTARY FIGURE AND TABLE LEGENDS


