# Supplementary material for: Swiprosin-1 stimulates cancer invasion and metastasis by increasing the Rho family of GTPase signaling
Source: Oncotarget. 2015 Apr 11;6(15):13060–71. doi: 10.18632/oncotarget.3637 (PMC4536999; doi:10.18632/oncotarget.3637)
Supplement: Supplementary file 1 [file oncotarget-06-13060-s001.pdf]

SUPPLEMENTARY FIGURE AND TABLE LEGENDS

A

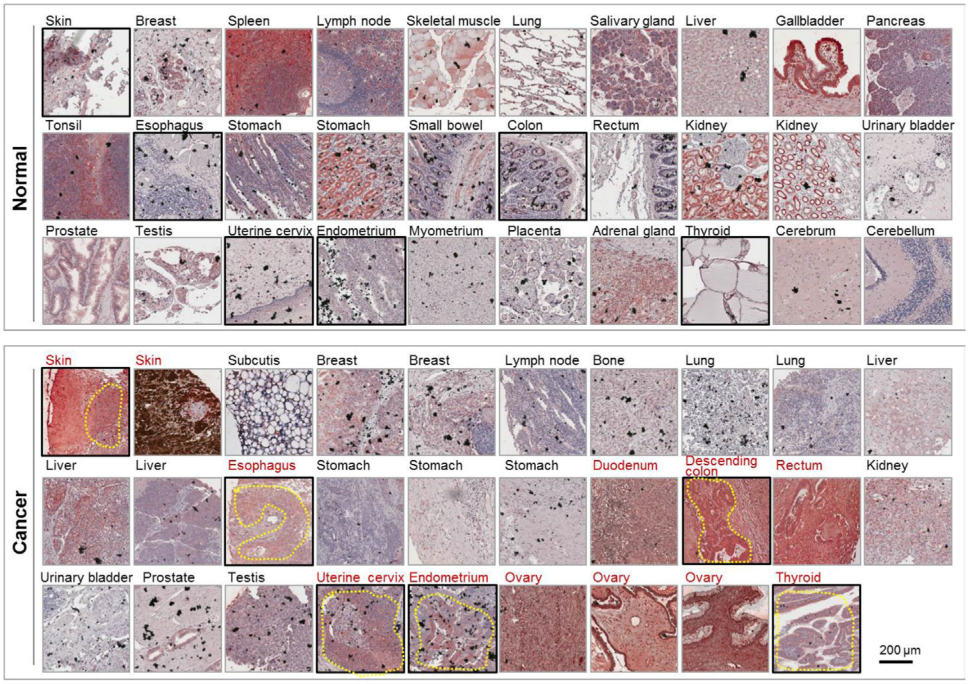

B

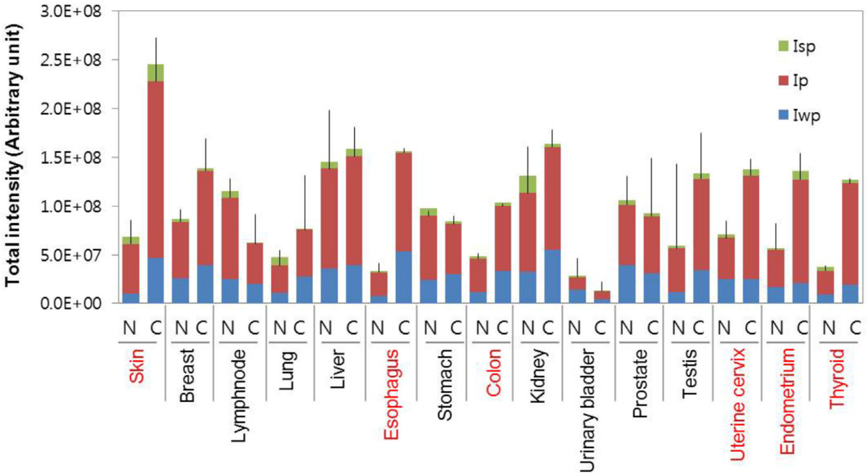

Supplementary Figure S1: Analysis of Swiprosin-1 expression in tissue microarray of human cancer. A. Tissue microarray containing 30 normal and 29 cancer tissue sections from human patients was stained with Swiprosin-1 antibody or subjected to H&E. Representative results from two independent experiments are displayed. Images in Figure 1B are marked in black boxes with bold lines, and tumor regions are indicated with yellow dotted lines. **B.** Expression patterns of Swiprosin-1 were compared in 15 matched TMA tissues. Intensity was quantified using the Positive pixel counts v.9.1 algorithm of AperioImageScope, and the algorithm computed the sum of intensity values for all weak positive pixels (IWP), modest positive pixels (IP), and strong positive pixels (ISP) in scanned images. Total intensity was calculated as the sum of IWP, IP and ISP. Stained images of tissues (red) are presented in Figure 1B.

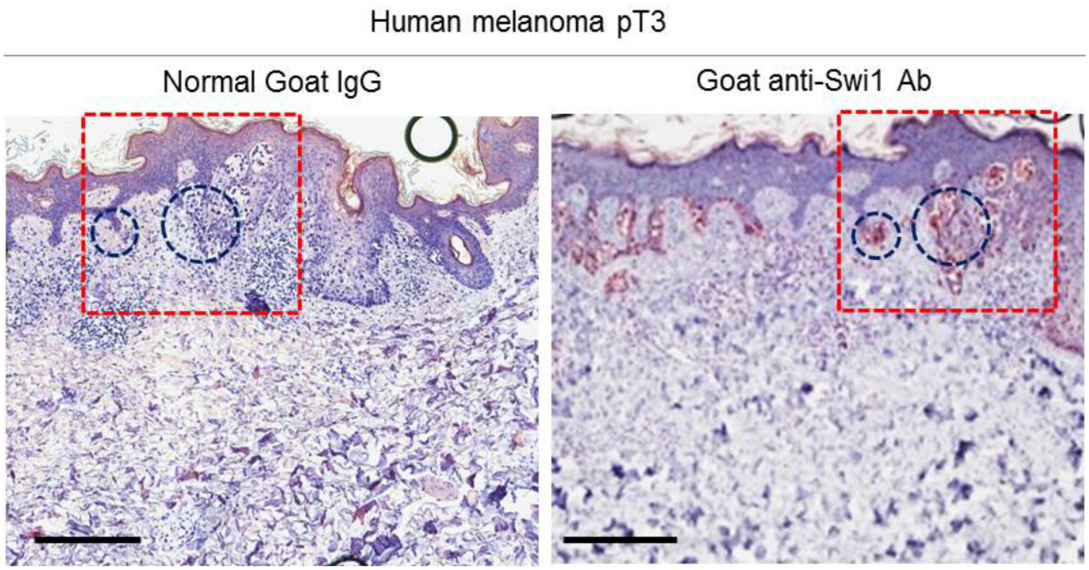

Supplementary Figure S2: Validation of anti-Swi-1 antibody specificity in human melanoma. To validate the specificity of anti-Swi-1 antibody in melanoma, tissues were incubated with normal goat IgG according to the standard immunohistochemistry protocol. *Bar* = 300 μm.

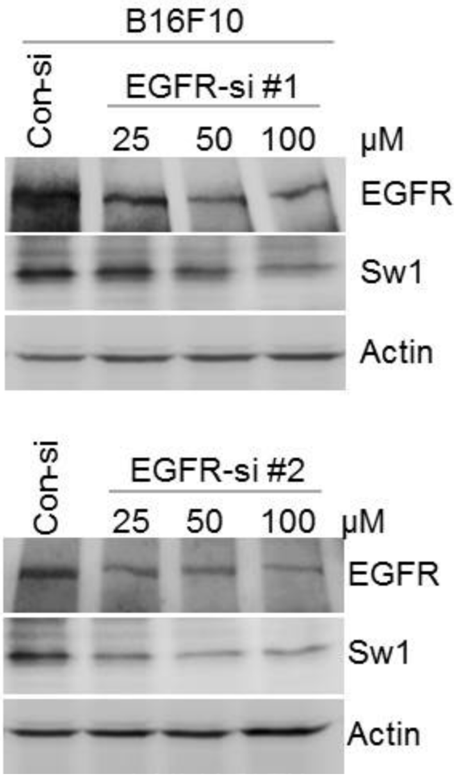

Supplementary Figure S3: Knockdown of EGFR inhibits Swiprosin-1 expression. B16F10 cells were transfected with EGFR siRNA in a dose-dependent manner, and knockdown of EGFR and Swiprosin-1 expression assessed.

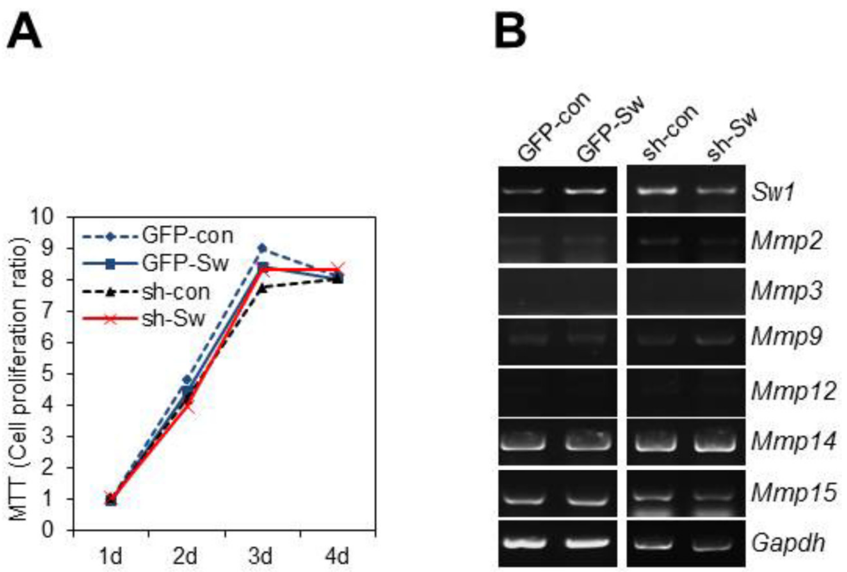

Supplementary Figure S4: Swiprosin-1 does not modulate proliferation and MMP expression in B16F10 melanoma cells. A. Swiprosin-1 overexpression or knockdown cells (0.5×10^4) cultured on 24-well plates for the indicated time-periods were subjected to the MTT assay. **B.** After 24 h of culture, total RNA was isolated and subjected to RT-PCR with specific primers for MMPs.

Supplementary Table S1: The information of human normal and cancer tissues

Organ	Diagnosis	[a)]Tissue type
Skin	Normal	2
Breast	Normal	3
Spleen	Normal	2
Lymph node	Normal	2
Skeletal muscle	Normal	2
Lung	Normal	3
Salivary gland, sublingual	Normal	2
Liver	Normal	2
Gallbladder	Normal	1
Pancreas	Normal	3
Tonsil	Normal	1
Esophagus	Normal	3
Stomach, antrum	Normal	3
Stomach, fundus	Normal	3
Small bowel	Normal	3
Colon	Normal	3
Rectum	Normal	3
Kidney, cortex	Normal	3
Kidney, Medulla	Normal	3
Urinary bladder	Normal	3

(*Continued*)

Organ	Diagnosis	a)Tissue type
Prostate	Normal	2
Testis	Normal	2
Uterine cervix	Normal	3
Endometrium	Normal	1
Myometrium	Normal	1
Placenta	Normal	1
Adrenal gland	Normal	2
Thyroid	Normal	3
Cerebrum	Normal	1
Cerebellum	Normal	1
Skin	Squamous cell carcinoma	4
Skin	Malignant melanoma	4
Subcutis	liposarcoma	4
Breast	ductal carcinoma *in situ*	4
Breast	infiltrating duct carcinoma	4
Lymph node	Hodgkin lymphoma	4
Bone	Osteosarcoma	4
Lung	Adenocarcinoma	4
Lung	Squamous cell carcinoma	4
Liver	cholangiocarcinoma	4
Liver	Hepatocellular carcinoma	4
Liver	Metastatic adenocarcinoma (from rectum)	4
Esophagus	Squamous cell carcinoma	4
Stomach	Adenocarcinoma	4
Stomach	Malignant lymphoma, diffuse large B cell	4
Stomach	Signet ring cell carcinoma	4
Duodenum	Gastrointestinal stromal tumor, malignant	4
Descending	Colon adenocarcinoma	4
Rectum	Adenocarcinoma	4
Kidney	Renal cell carcinoma	4
Urinary bladder	Transitional cell carcinoma	4
Prostate	Adenocarcinoma	4
Testis	Seminoma	4
Uterine cervix	Squamous cell carcinoma	4

(*Continued*)

Organ	Diagnosis	[a)]Tissue type
Endometrium	Adenocarcinoma	4
Ovary metastatic	Adenocarcinoma (from stomach)	4
Ovary mucinous	Cystadenocarcinoma	4
Ovary	Serous cystadenoma of low malignant potential	4
Thyroid	Papillary carcinoma	4

[a)]Normal and cancer tissues were obtained from different patients. The "tissue type" column includes the following categories:

1. normal tissue from non-cancer patients
2. normal tissue from cancer patients, but the cancer involves unrelated organs
3. normal tissue adjacent to the cancer
4. Cancer

LEGENDS FOR SUPPLEMENTARY MOVIES

(Supplementary movies are separately linked)

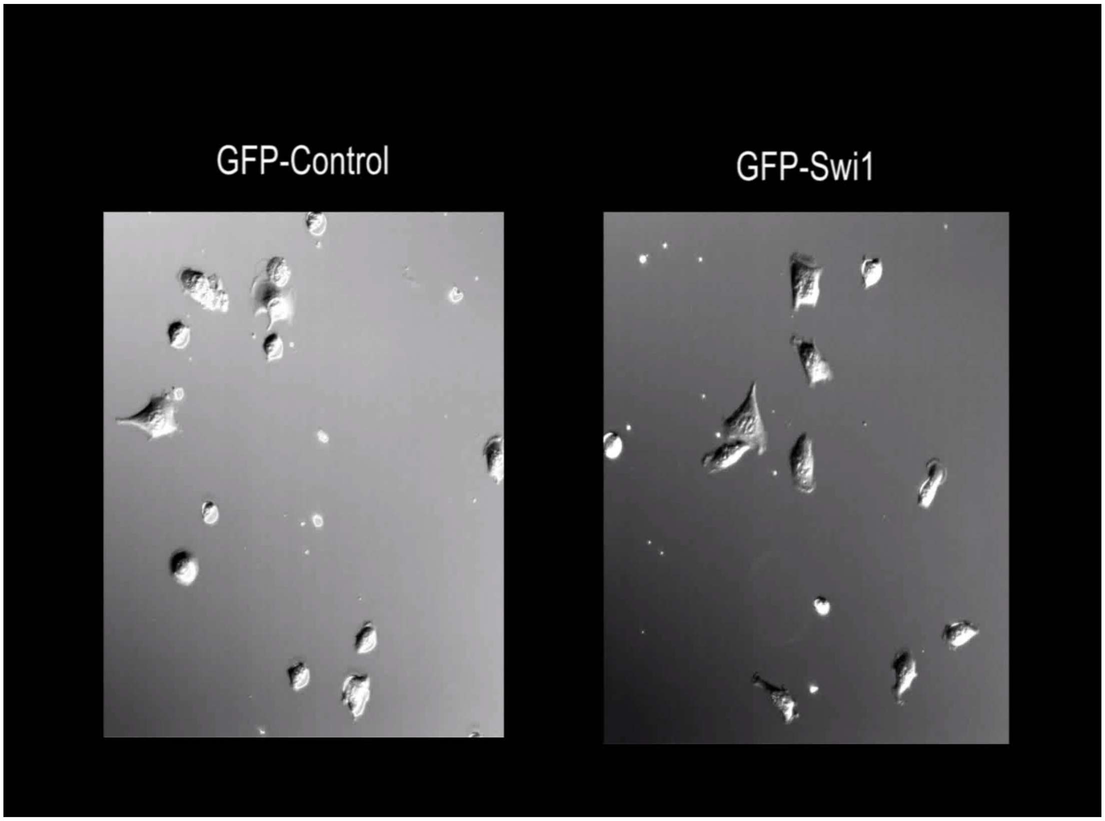

Supplementary MOVIE SM1: Motility of GFP-control and GFP-Swiprosin-1-expressing cells. Time-lapse images of B16F10 cells stably expressing GFP or GFP-swiprosin-1 were captured every 10 min for a total of 9 h. Analysis of cell migration velocity via nuclear tracking is shown in Figure 4E.

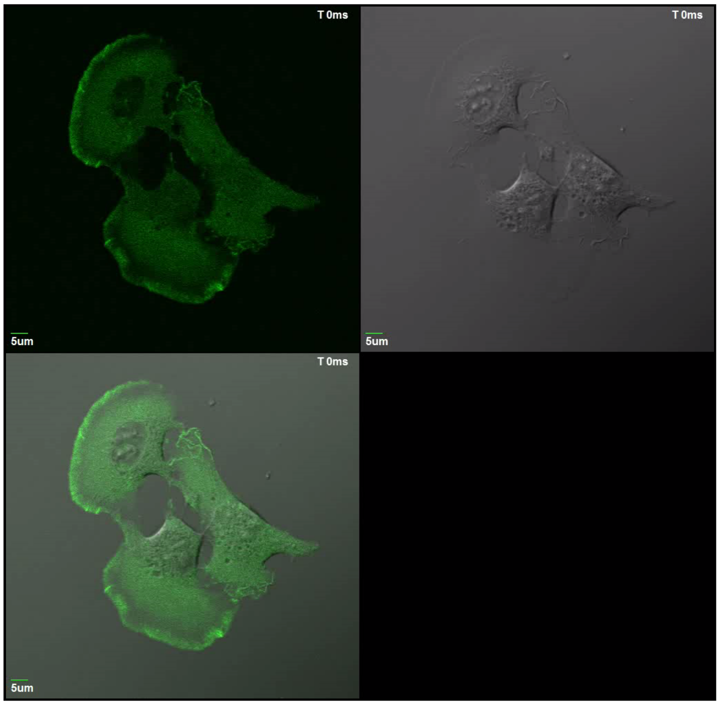

Supplementary MOVIE SM2: Translocation of GFP-Swiprosin-1 in B16F10 cells. Time-lapse fluorescence images of GFP-Swiprosin-1-expressing cells were captured at 5 sec intervals for 10 min using confocal microscopy. Images obtained at the selected time-points are presented in Figure 5A.

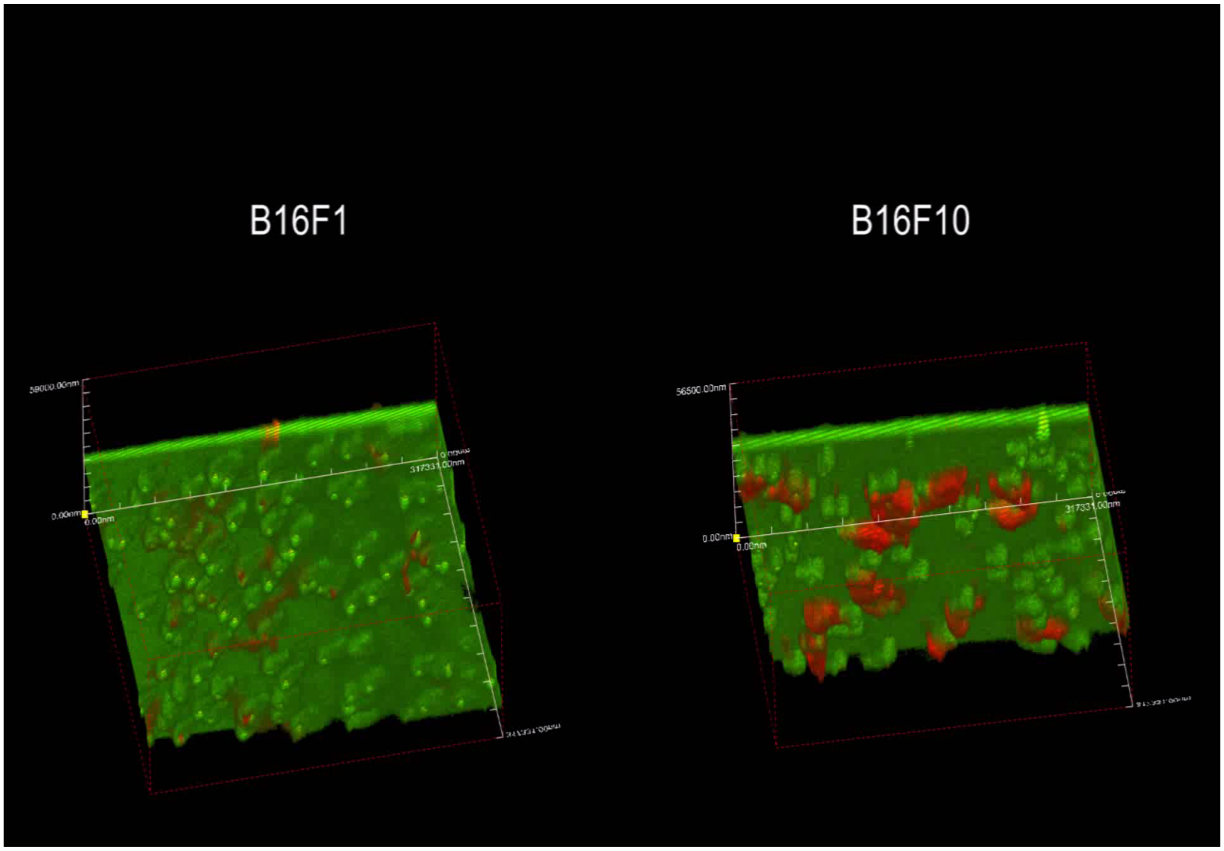

Supplementary MOVIE SM3: 3D animation of vertical section images of cells on a FITC-gelatin-coated transwell membrane. Angle rotation movies corresponding to Figure 5E (B16F1 and B16F10).

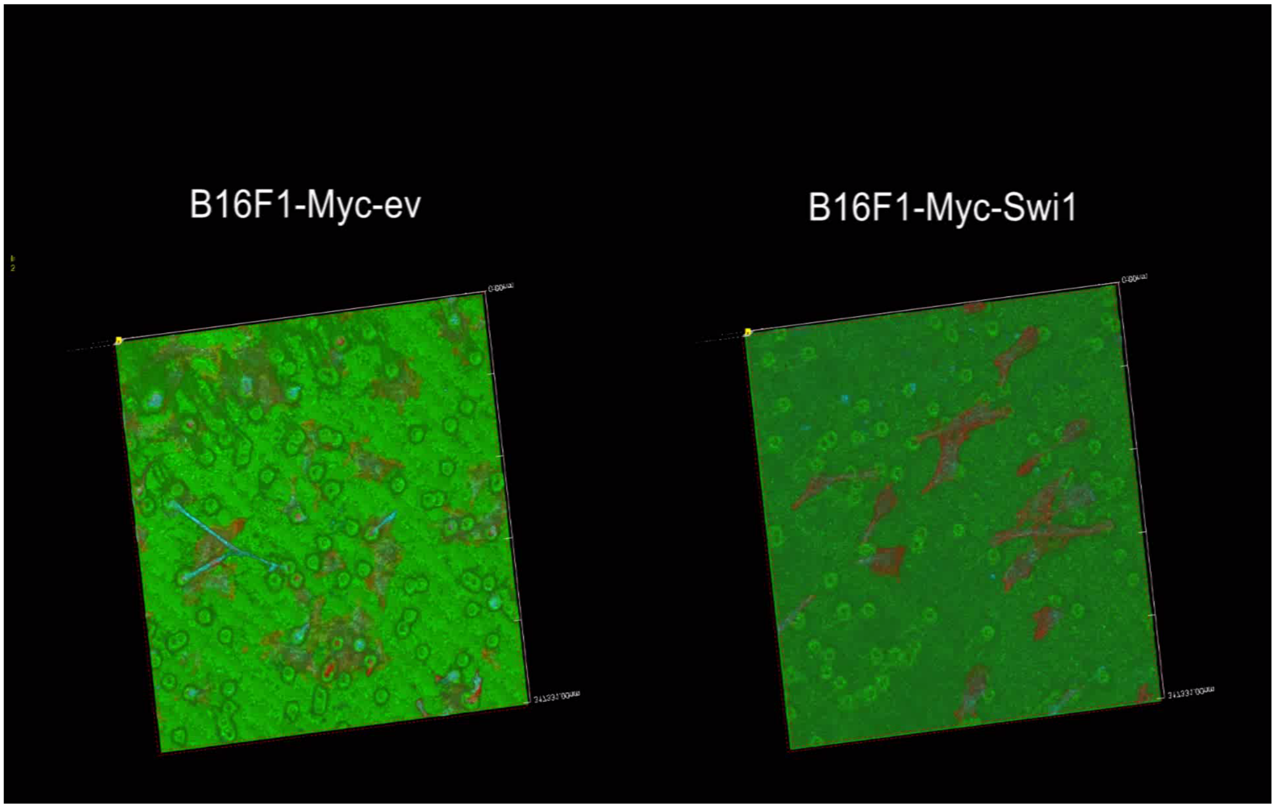

Supplementary MOVIE SM4: 3D animation of vertical section images of cells on a FITC-gelatin-coated transwell membrane. Angle rotation movies corresponding to Figure 5F. B16F1 cells transfected with Myc-empty vector (B16F1-Myc-ev) and Myc-Swiprosin-1 (B16F1-Myc-Sw1).
